# Engineering Photolabile Cyclopentane‐Fused Coumarins: From One‐Pot Synthesis to Mechanistic Study for Visible Light‐Triggered Photolysis

**DOI:** 10.1002/asia.202500760

**Published:** 2025-07-29

**Authors:** Jau‐Tien Lin, Hsuan‐Yu Lin, Tai‐Chung Lo, Chih‐Ling Lin, Yi‐Hsien Li, Wei‐Hao Wu, Ting‐Yi Hsieh, Shun‐Yuan Luo, Chih‐Chien Chu

**Affiliations:** ^1^ Department of Medical Applied Chemistry Chung Shan Medical University Taichung 402 Taiwan; ^2^ Department of Chemistry National Chung Hsing University Taichung 403 Taiwan; ^3^ Department of Medical Education Chung Shan Medical University Hospital Taichung 402 Taiwan

**Keywords:** Coumarin, Cyclopentane fusion, Fluorescence tracing, Photocages, Photo‐elimination

## Abstract

A series of cyclopentane‐fused coumarins were synthesized via a one‐pot Heck–Aldol annulation cascade from diethylaminocoumarin derivative and evaluated as visible light‐responsive photocages. Compared to conventional coumarin photocages, these fused systems exhibited substantially different photolysis behavior. UV–vis spectroscopic analysis showed distinctive evolution of absorption profiles during photolysis, with pronounced bathochromic shifts and emergence of new bands at 405–415 nm, contrasting sharply with the minimal spectral changes seen in conventional photocages. Kinetic studies revealed that the cyclopentane‐fused derivatives underwent significantly slower photorelease (*k* = 0.2106 h^−1^) and lower photolytic quantum yields (*Φ*_u_ = 1.0 × 10^−3^) than nonfused analogs, with pronounced solvent dependence not observed in traditional photocages. The spectral transformations, together with HPLC and LC‐MS data, suggested a photo‐elimination pathway involving heterolytic C─O bond cleavage followed by β‐proton elimination rather than the conventional photo‐S_N_1 mechanism, with the photoproduct identified as a cyclopentene‐fused structure (m/z 314.1). Fluorescence spectroscopy demonstrated a direct correlation between emission intensity decrease and uncaging progress, providing a convenient real time monitoring method. The contrasting kinetic profiles and spectroscopic signatures between rapid‐release conventional photocages and sustained‐release fused systems offer complementary tools for applications requiring different payload delivery rate, expanding the photochemical toolbox for controlled release applications.

## Graphical abstract

Cyclopentane fusion in coumarin photocages transforms their behavior from rapid S_N_1‐mediated release (minutes) to a slower elimination pathway (hours), accompanied by dramatic spectral changes upon photolysis, providing complementary tools for controlled‐release applications requiring different payload delivery rates.

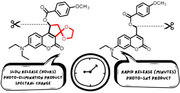

## 1 Introduction

**Scheme 1:**
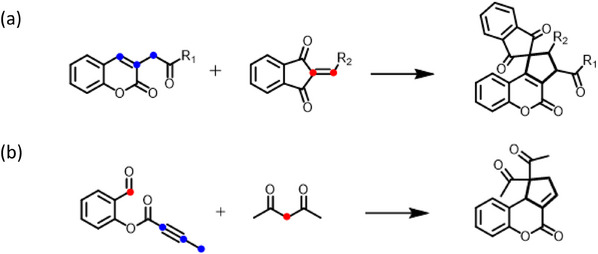
Synthetic approaches for cyclopentane‐fused coumarins: a) [3 + 2] cycloaddition of 3‐homoacyl coumarin with dipolarophiles; b) one‐pot [3 + 2] annulation of 2‐formylphenyl alkynoates with activated methylene compounds.

The carbocyclic and heterocyclic‐fused coumarin derivatives exhibit a diverse range of biological and pharmacological activities; the fusion of coumarins with versatile cyclic building blocks not only alter their physicochemical properties but also typically enhance their biological activities.^[^ ^1^, ^2^, ^3^, ^4^, ^5^, ^6^ ^]^ While there has been substantial work on developing synthetic approaches for heterocyclic‐fused coumarins, carbocyclic analogs, such as cyclopentane‐fused coumarins, have received less exploration, with naturally occurring aflatoxin derivatives being notable representatives.^[^ ^7^, ^8^ ^]^ As shown in Scheme 1a,b, two notable examples illustrate facile construction of cyclopentane‐fused ring derived from the C3─C4 bond of coumarin. The first approach utilizes [3 + 2] cycloaddition of α,β‐unsaturated carbonyl compounds and conjugated dienes with 1,3‐dipolar precursor 3‐homoacyl coumarin.^[^ ^9^ ^]^ Yang and coworkers also proposed an improved one‐pot [3 + 2] annulation of 2‐formylphenyl alkynoates with activated methylene compounds to construct cyclopentene‐fused dihydrocoumarin via a domino sequence.^[^ ^10^ ^]^ The strategies involve the cycloaddition process to generate the fused ring through either direct coupling of coumarin derivatives with dipolarophiles or a cascade construction of coumarin and cyclopentane rings.^[^ ^11^, ^12^, ^13^ ^]^

**Scheme 2:**
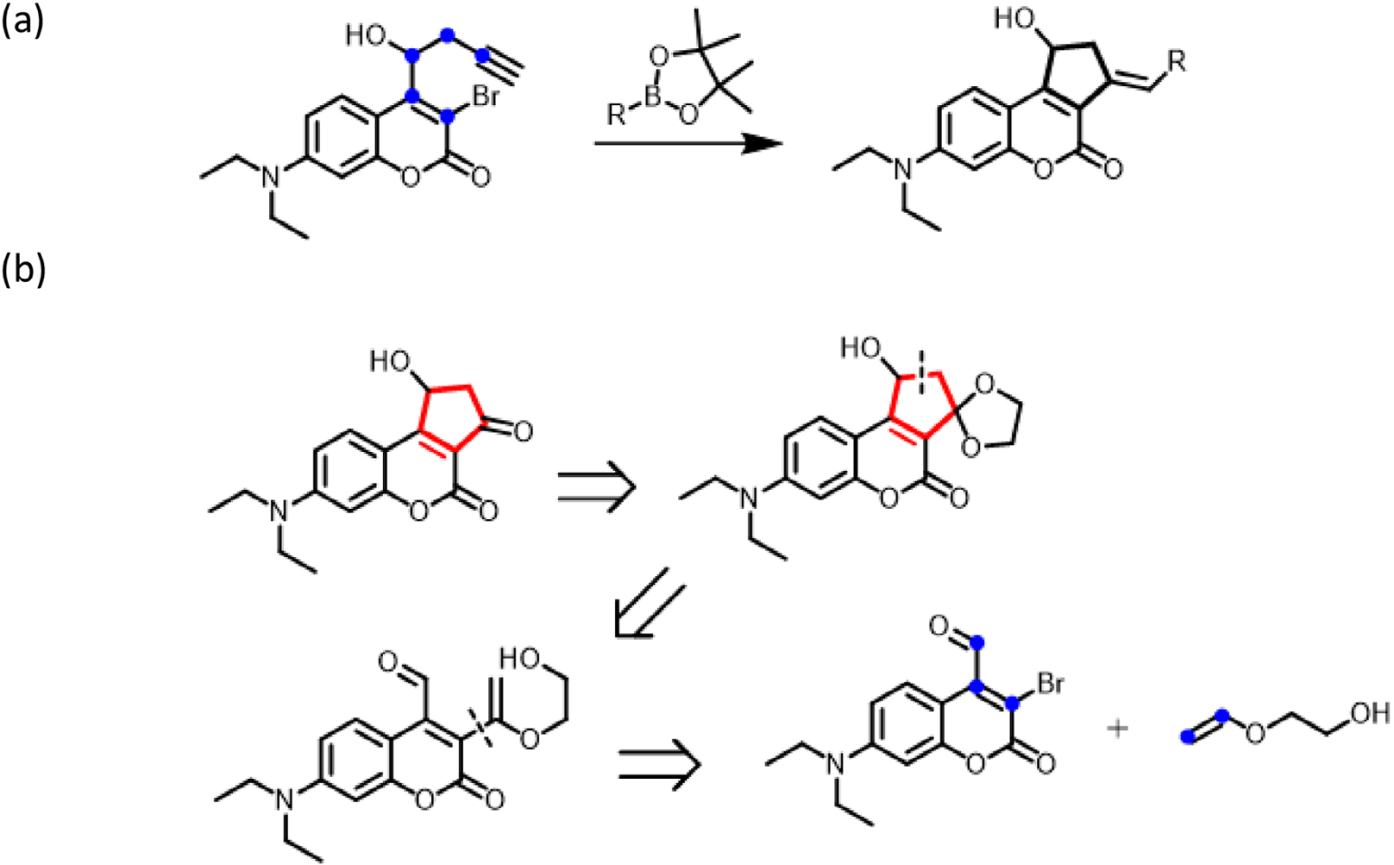
a) Specht's cyclocarbopalladation approach for constructing cyclopentane‐fused coumarins. b) Retrosynthetic analysis for the cyclopentane‐fused coumarins with a C3 ketone via Heck–Aldol annulation.

An alternative approach for constructing the cyclopentane‐fused coumarins involves the intramolecular cyclization of the C3 and C4‐disubstututed coumarin derivatives. Specht and coworkers have proposed a cyclization strategy using 3‐bromo‐4‐(1‐hydroxybut‐3‐ynyl)‐substituted coumarin as the starting molecule via the 5‐*exo*‐dig cyclocarbopalladation (Scheme 2a).^[^ ^14^ ^]^ This methodology, coupled with a domino process involving cyclocarbopallation followed by Suzuki–Miyaura or Sonogashira cross‐coupling reactions, leads to a π‐extended coumarin system through the *exo* double bond of the fused cyclopentane ring. The photolytic properties of these fused coumarins were investigated, showing remarkable quantum yields in the photoinduced uncaging process.

Feringa and colleagues further advanced our understanding of photolysis efficiency by demonstrating that stabilization of intermediate cations substantially improves the efficiency of photolabile chromophores.^[^ ^15^, ^16^ ^]^ A critical factor in this process is preventing recombination of the contact ion pair (CIP) following photo‐induced dissociation. The stabilization of cationic intermediates follows the order allyl > 3 ° > 2 ° > 1 °, which effectively raises the energy barrier of the recombination process and systematically enhances photolysis quantum efficiency. This concept aligns with the proposed photolytic mechanism for the cyclopentane‐fused coumarins (Scheme 2a), involving a photoinduced heterolytic cleavage of the C─O bond and significant formation of stable secondary (2°) carbocation intermediates on the cyclopentane ring. Motivated by these findings and seeking to explore new synthetic routes, we propose a one‐pot Heck–Aldol‐type annulation cascade for constructing the fused five‐membered ring using 3‐bromo‐4‐formyl‐substituted diethylaminocoumarin (DEAC) as the starting molecule.^[^ ^17^, ^18^ ^]^ Scheme 2b depicts the retrosynthetic analysis for the cyclopentane‐fused coumarins with a C3 ketone as the external electron‐withdrawing group; the steps involve a ketal protection by ethylene glycol (EG), followed by the bond formation via intramolecular aldol reaction and α‐regioselective Heck coupling reaction. Notably, expanding on a modified condition developed by Xiao and coworkers, the use of EG as a solvent is anticipated to facilitate the Heck–Aldol annulation, yielding 3‐hydroxy‐1‐indanones.^[^ ^19^ ^]^ EG not only acts as a promoter for regioselective Heck coupling reactions but also as an activator for the carbonyl oxygen through hydrogen bonding during the aldol‐type cyclization. This intramolecular cyclization strategy not only provides an alternative to the previously mentioned cycloaddition approach but also offers opportunities for installing various functional groups on the fused cyclopentane ring, allowing for further derivatization and tuning of the photoresponsive properties of the coumarin compounds.

## 2 Results and Discussion

### 2.1 Materials Synthesis

**Figure 1:**
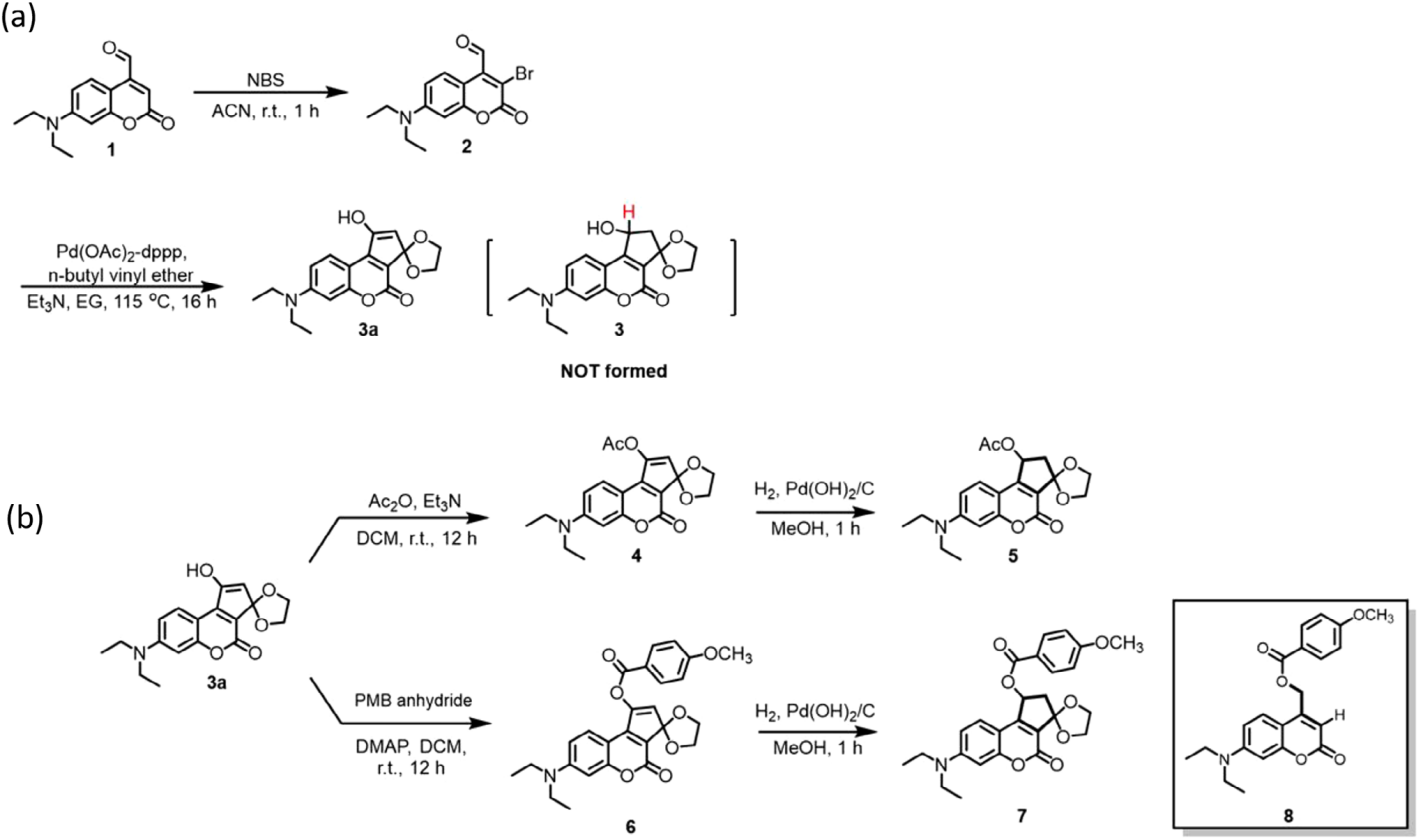
Synthetic route for cyclopentane‐fused coumarins: a) regioselective bromination followed by Heck coupling to produce intermediate 3a. b) Protection and reduction of 3a to yield compounds **4–7**.

The starting molecule **1**, a DEAC derivative with a C4‐derived cabaldehyde was synthesized from coumarin 460 following the published procedure.^[^ ^20^ ^]^ As shown in Figure 1a, regioselective bromination of C3 position producing new compound **2** was then successfully performed using *N*‐bromosuccinimide (NBS) in the presence of ammonium acetate as the catalyst. A cascade Heck coupling and intramolecular aldol reaction was conducted based on the EG‐promoted annulation protocol. However, the effort for optimizing the reaction only led to compound **3a** in a 14% yield instead of the expected cyclization product **3** (Supporting Information). According to the previous reports, we assume that the first step is Pd‐catalyzed olefination of compound **2** and *n*‐butyl vinyl ether (BVE), and α‐regioselectivity for furnishing branched olefins at C3 can be carried out using electron‐rich BVE in EG as the alcohol solvent. We found that uncyclized Heck product with a ketal group can be isolated only as the temperature was lower than 80 °C. Therefore, we concluded that the butyl ether group was replaced by EG before cyclization, so the ketal product is a significant intermediate for this one‐pot annulation, which is agreed with Hallberg and coworkers’ report.^[^ ^18^ ^]^ The second step is the intramolecular nucleophilic attack between the branched vinyl ether and C4 aldehyde group in the presence EG as the carbonyl activator. It is also believed that the electron‐rich feature of the vinyl ether group should facilitate this intramolecular process. Forming a C═C double bond but keep the OH group at the cyclopentyl ring excluded Elcb mechanism of the aldol condensation. Therefore, we hypothesized a β‐hydride elimination‐like process in the presence of Pd catalyst to generate corresponding ketone group at C4’ position.^[^ ^21^, ^22^, ^23^ ^]^ Eventually, a tautomerization of ketone to enol leads to compound **3a**, which seems to be a thermodynamically‐driven product due to π‐conjugation skeleton of the fused cyclopentyl ring and coumarin.

**Figure 2:**
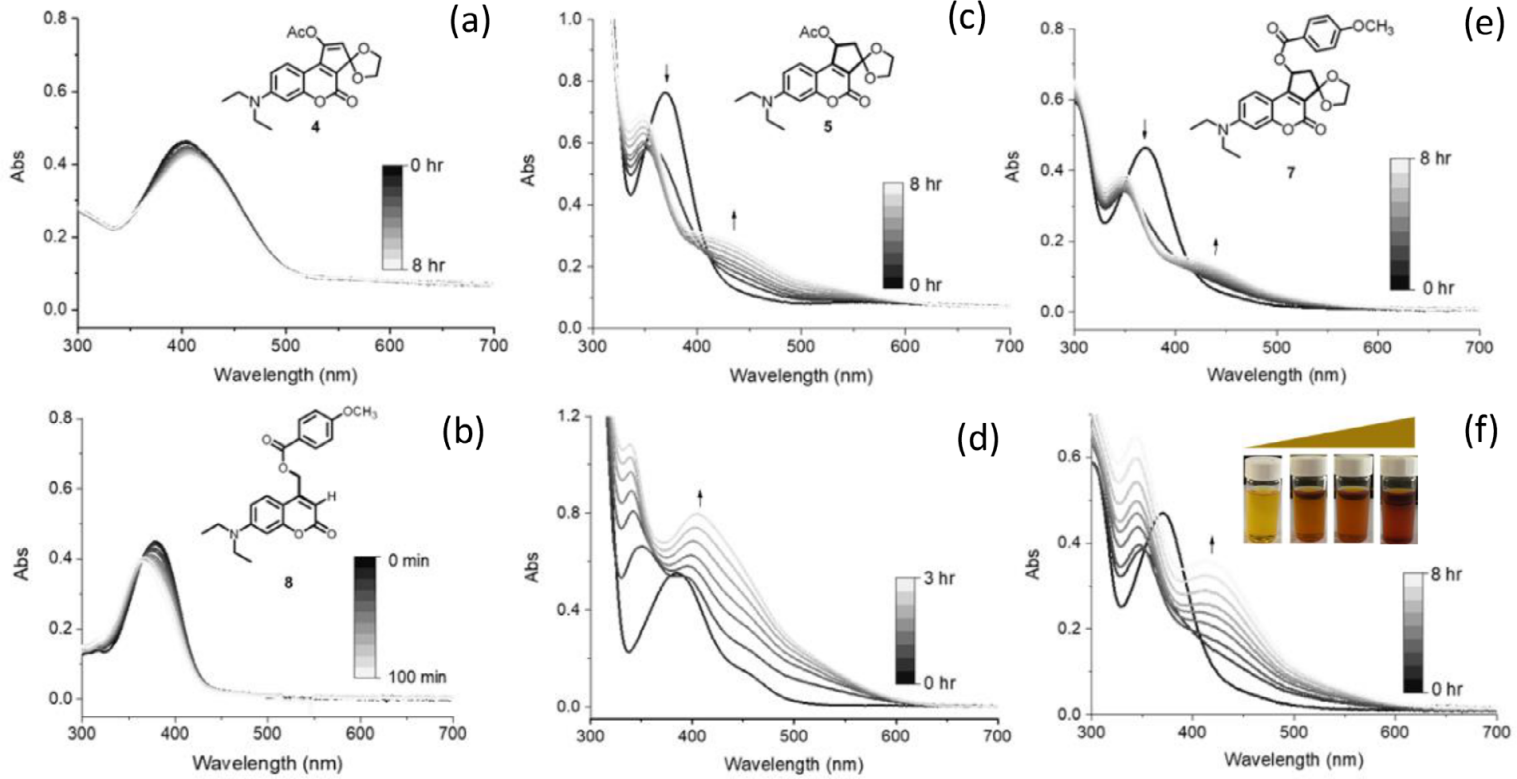
UV–vis absorption spectra of coumarin derivatives during photolysis: a) compound **4** in H_2_O/MeOH (1:9 v/v); b) reference compound **8** in H_2_O/MeOH (1:9 v/v); c) compound **5** in H_2_O/MeOH (1:9 v/v); d) compound **5** in PBS/MeOH; e) compound **7** in H_2_O/MeOH (1:9 v/v); f) compound **7** in PBS/MeOH with visual observation of color change.

To obtain the Heck–Aldol product with a C─C bond at cyclopentyl fused ring, as shown in Figure 2b, the OH group at C4’ position of compound **3a** was first protected by an acetyl (Ac) group to yield **4** quantitatively. Then a catalytic hydrogenation was performed in H_2_ atmosphere of 5 bar to produce target compound **5** within 1 h. Based on this successful protocol, the OH group was also modified by a *p*‐methoxybenzoyl (PMB) group through base‐promoted esterification to give compound **6**, followed by hydrogenation to produce compound **7**. To investigate the effect of the fused ring structure on photolytic behavior, compound **8** with a PMB payload was also synthesized as a reference compound.^[^ ^24^ ^]^

### 2.2 Photophysical Properties and Photolysis Study

The photophysical properties of the coumarin derivatives are presented in Table 1. Compound **1**, bearing a C4‐substituted carbonyl group, exhibits the most red‐shifted absorption maximum (*λ*_max_ = 442 nm) among the series. This bathochromic shift can be attributed to the electron‐withdrawing nature of the carbonyl group, which enhances donor–acceptor (D‐π‐A) character of the coumarinyl system.^[^ ^25^ ^]^ Upon cyclization to form compounds **3a**, **5**, and **7**, the absorption maxima are blue‐shifted to the 360–380 nm region. This hypsochromic shift suggests a reduction in the D‐π‐A character, likely due to the rigidification of the molecular framework imposed by the cyclic constraints. Particularly, while compounds **3a**, **5**, and **7** exhibit similar absorption wavelengths, their molar extinction coefficients (*ε*_max_) vary significantly. Compared to **3a**, the ε values of **5** and **7** are decreased by approximately 30% and 50%, respectively. These diminished extinction coefficients imply a reduced probability for the electronic transitions in the cyclized derivatives, which could be attributed to conformational effects or changes in the electronic delocalization pathways. Turning to the emission properties, compound **1** displays the most red‐shifted fluorescence maximum (*λ*_max_ = 567 nm), albeit with a very weak intensity, as indicated by the low quantum yield (*Φ*_f_). Upon cyclization to **3a**, the *Φ*_f_ value modestly increases to 0.14, suggesting that the rigid cyclic structure may facilitate the radiative deactivation pathways. Compare to the reference compound **8**, the ring‐fused compound **5** and **7** show similar absorption and emission wavelengths, but lower absorption coefficients. Notably, these photolabile compounds exhibit moderate *Φ*_f_ of approximately 0.5–0.7. This combination of blue fluorescence and reasonable quantum efficiencies renders target compounds suitable for applications that involve monitoring photolytic processes through fluorescence techniques.^[^ ^26^ ^]^

**Table 1:** Photophysical properties of coumarin derivatives in THF.

Compound	λ_max_ (nm)	ε_max_ (10^4^ M^−1^ cm^−1^)^a)^	λ_max_^fl^ (nm)	*Φ*_f_ ^b)^
**1**	440	1.19	570	0.01
**3a**	380	1.33	440	0.14
**5**	370	0.89 (0.55)	430	0.56
**7**	365	0.61 (0.49)	425	0.58
**8**	370	1.81 (1.66)	440	0.72

^a)^ Extinction coefficients in parentheses were measured in methanol/H_2_O (9:1 v/v) at 405‐nm.

^b)^ Coumarin 337 used as calibration standard. (*Φ*_f_ = 0.94 in acetone).

The photolytic experiments for these coumarin derivatives in methanol and water mixture (9:1 v/v) were performed by 405‐nm LED irradiation, which is less harmful to the biological system. Then the change in absorption spectra for the coumarin compounds were recorded. Based on the absorption spectra shown in Figure 2, there are some distinct behaviors observed for the ring‐fused coumarin derivative under irradiation with LED light. First, compound **4** with a C═C bond at fused cyclopentyl ring exhibits minimal changes in absorption profiles over the 8‐h irradiation period (Figure 2a). The initial sharp absorption peak around 380 nm persists with only minor broadening and slight bathochromic shift, indicating good photostability of coumarin skeleton under these conditions. However, compound **5** with a C─C bond at the fused ring reveals a markedly different trend (Figure 2c). Upon irradiation, a new broad absorption band emerges in 400–500 nm, gradually increasing in intensity over time. Concurrently, the original peak at 370 nm disappears in 1 h, suggesting the depletion of the starting material and the formation of a photoproduct species. The observed spectral changes upon irradiation provide crucial insights into the photochemical mechanism. A conventional photo‐S_N_1 pathway can be ruled out based on the significant alterations in the absorption spectra. In a typical photo‐S_N_1 reaction for reference compound **8**, the photoproduct would retain a π‐conjugation structure similar to the starting material, resulting in minimal changes to the absorption profile (Figure 2b).^[^ ^24^ ^]^ Alternatively, this spectral evolution strongly suggests a more complex photochemical process, likely involving a photo‐elimination mechanism for the coumarin derivative proposed by Specht and coworkers.^[^ ^14^ ^]^ The two‐step mechanism would involve initial heterolytic cleavage of a C─O bond, followed by the elimination of a β‐proton and the generation of a C═C bond. The resulting photoproduct, characterized by extended π‐conjugation, would exhibit distinctly different optical properties from the starting molecule. This extended conjugation may explain the observed bathochromic shift and broadening of the absorption band.

The absorption spectra of compound **5** recorded in a phosphate‐buffered saline (PBS) solution with methanol reveals distinct photochemical behaviors, highlighting the influence of the solvent environment on the photoreactivity. Figure 2d exhibits a more rapid and pronounced spectral transformation; the initial peak at 370 nm diminishes significantly within 30 min of irradiation, whereas a new broad absorption band emerges (*λ*_max_ = 405 nm), rapidly increasing in intensity within 3 h. The accelerated rate of photochemical transformation observed in the PBS/methanol system could be attributed to the presence of ionic species facilitating the abstraction of β‐proton in the proposed elimination pathway, thereby promoting the formation of the C═C double bond and the generation of the photoproduct species observed in the absorption spectra. In contrast, in the water/methanol solvent system, the deprotonation step may be less favored, leading to a slower rate of photoproduct formation. Generally, the ionic species in the PBS solution could act as mild bases driving the photo‐elimination more efficiently. As shown in Figure 2e,f, compound **7** also exhibits rapid spectral change in PBS/methanol upon light irradiation, and the photoproduct species with bathochromic shift to *λ*_max_ = 415 nm was constantly accumulated within 8 h. It can be found that the pale yellow solution of the cyclized compounds became brown‐to‐orange under light exposure, which is related to the shift of the absorption spectra from UV to visible‐light wavelength. Based on the observation of color change, the photolytic process accompanied with releasing of the PMB payload can be readily traced.

### 2.3 Kinetic Studies and Mechanistic Investigation of the Photolytic Release

**Figure 3:**
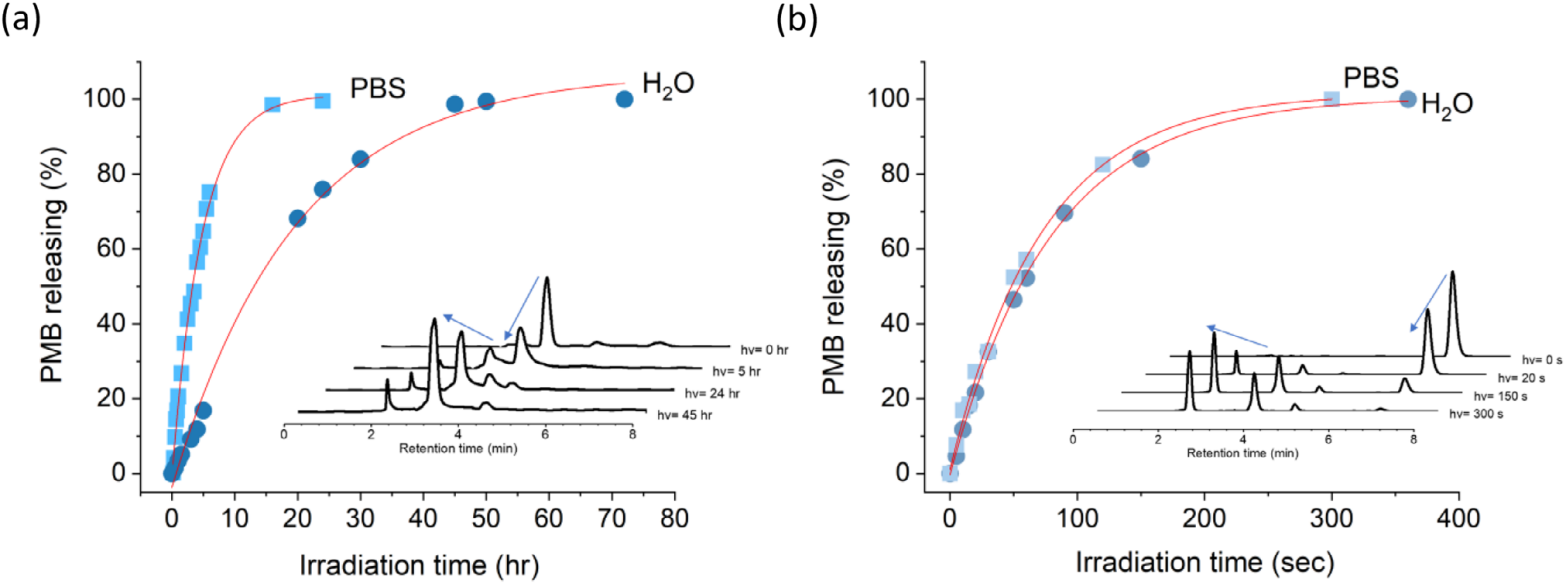
Time‐dependent photolytic release of PMB in different solvent systems: a) first‐order release kinetics for compound **7** in H_2_O/MeOH and PBS/MeOH under 405 nm LED irradiation; b) first‐order release kinetics for reference compound **8** showing solvent‐independent behavior. The insets show the evolution of the HPLC chromatograms for two compounds upon light irradiation in H_2_O/MeOH solutions.

The releasing of PMB payload in aqueous solutions under light excitation was further analyzed by a reversed‐phase HPLC using methanol and water mixture (9:1 v/v) as a mobile phase. The chromatograms reveal that gradual depletion of the compound **7** at *t* = 4.6 min over time upon irradiation, with the formation of the PMB payloads observed as a distinct peak at *t* = 3.3 min (The inset in Figure 3a). A quantitative analysis of the release kinetics was performed by monitoring the peak areas corresponding to the released PMB in HPLC chromatograms, and then calibrating by the PMB standard with known concentrations. As shown in Figure 3a, the PMB molecules were completely released within approximately 40 and 15 h in H_2_O/methanol and PBS/methanol systems, respectively. Furthermore, the releasing profiles are well‐fitted by the first‐order kinetics, enabling the determination of the rate constants (*k*) in two systems. The kinetic data reveals a solvent‐dependent behavior in the photoinduced PMB releasing, with the *k* = 0.051 h^−1^ in the aqueous environment and *k* = 0.2106 h^−1^ in the PBS buffer solution, representing an approximately fourfold increase in the latter. This result is consistent with the observation in UV–vis absorption analysis, suggesting that the substantial rate enhancement in the PBS environment can be attributed to the presence of ionic components, such as phosphate ions, which likely play a crucial role in facilitating the proposed photo‐elimination pathway. Additionally, the ionic environment could influence factors, such as excited‐state dynamics, solvent cage effects, or stabilization of carbocation intermediates, further contributing to the accelerated kinetics observed in the PBS solution. Interestingly, despite the difference in rate constants, both kinetic profiles exhibited first‐order behavior, suggesting that the rate‐determining step remains the same in both solvent systems. This observation implies that the solvent environment primarily affects the kinetic favorability of the reaction, rather than altering the underlying mechanism.

The photolytic release profiles of **8** exhibited markedly different characteristics compared to compound **7** (Figure 3b). While maintaining first‐order kinetics in both H_2_O/methanol and PBS/methanol solutions, compound **8** showed nearly identical release rates regardless of the solvent environment with the rate constant *k* = 4.97 × 10^1^ h^−1^. In addition, compound **8** showed notably faster release kinetics compared to compound **7**. This dramatic difference in reaction rates‐with compound **8** proceeding approximately 2 orders of magnitude faster than compound **7**‐provides strong evidence for distinct photolytic mechanisms. Given that compound **8** operates through a well‐established photo‐S_N_1 mechanism, the contrast between compounds **7** and **8** in their response to PBS strongly supports our hypothesis that compound **7** proceeds through a distinct photo‐elimination pathway rather than the conventional photo‐S_N_1 mechanism typical of coumarin‐based photocages.

The photo‐induced uncaging quantum yield (*Φ*_u_) can be calculated by a comparative method referring to the reference compound.^[^ ^27^ ^]^ The reported *Φ*_u_ value for the reference compound **8** is 7.0 × 10^−2^, and eventually, the calculated *Φ*_u_ values for compound **7** in H_2_O/methanol and PBS/methanol systems were found to be 2.5 × 10^−4^ and 1.0 × 10^−3^, respectively. These calculations were based on the extinction coefficients measured in aqueous methanol solutions at 405 nm (Table 1). The photo‐induced uncaging efficiency of the fused coumarins is lower than that of the reference compound, with compound **7** showing approximately 2 orders of magnitude lower quantum yield compared to compound **8**. Considering the lower absorption coefficient of our cyclic‐fused coumarin derivatives, the overall reduced photolysis rate constants are understandable. Because the only structural difference between compounds **7** and **8** is the cyclopentane‐fused structure, current results indicate that the fused ring not only causes a decrease in the molar absorption coefficient of the coumarin due to disruption of the π‐conjugated planar structure but also leads to increased molecular stability upon irradiation, resulting in lower photolysis efficiency. Although both compounds follow first‐order kinetics, their different releasing kinetics suggests distinct photolytic pathways. The lower photolysis efficiency may be attributed to the geometric conformational changes required for the elimination reaction, making the elimination pathway slower than S_N_1, particularly evident in cyclic structures where achieving the antiperiplanar arrangement necessary for β‐proton elimination may be more challenging. Based on these observations, removing the ketal group at the C3 position of compound **7** to obtain the sp^2^ ketone might increase molecular planarity and enhance the thermodynamic stability requirements for the β‐proton elimination process. However, our attempts to remove the ketal protecting group of compound **7** through acidic treatment were unsuccessful, indicating that our fused coumarin compounds exhibit good stability under acidic conditions.^[^ ^8^ ^]^ Therefore, alternative deprotection strategies need to be explored.

**Figure 4:**
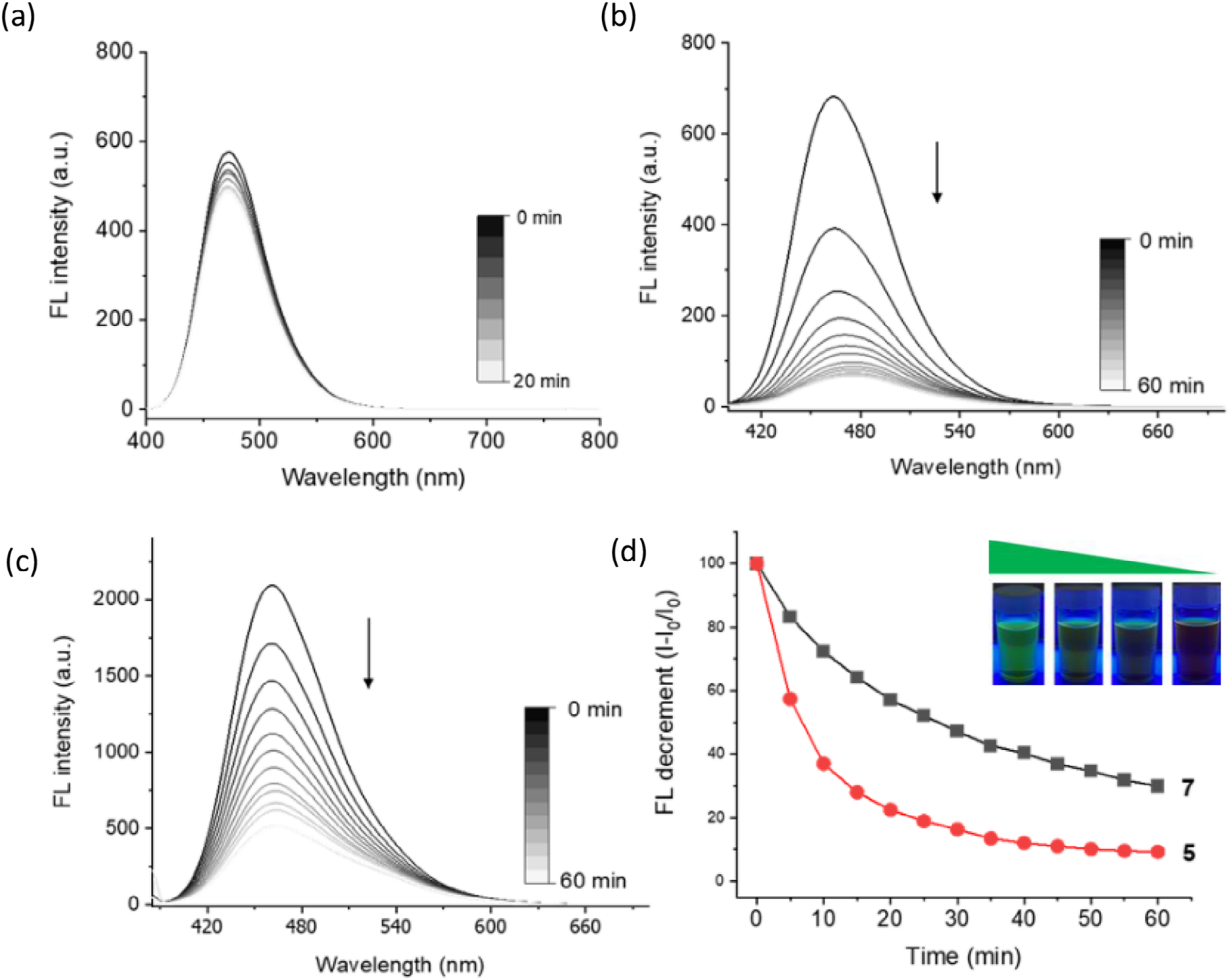
Fluorescence spectral changes during photolysis (405 nm LED). a) Minimal changes for reference compound **8**. b) Significant decrease in fluorescence intensity for compound **5**. c) Fluorescence intensity reduction for compound **7**. d) Comparative plot of fluorescence intensity decrease for compounds 5 and 7 with visual observation of emission color change.

As shown in Figure 4a, the photolytic behavior of reference compound **8**, which proceeds through a photo‐S_N_1 mechanism, showed minimal spectral changes during irradiation, with fluorescence spectra remaining largely unaltered over the course of photolysis. This spectral stability is consistent with its photo‐S_N_1 pathway reported in the literatures.^[^ ^28^, ^29^ ^]^ In contrast, compound **5** and **7** exhibited significant spectroscopic changes upon irradiation; as previously discussed, its UV–vis absorption spectrum showed a characteristic evolution with a decrease at 380 nm and the concurrent emergence of a red‐shifted band at 420 nm (Figure 2c–f). Furthermore, as shown in Figure 4b,c, the decrease in fluorescence intensity over time upon irradiation can be correlated with the photoinduced release of the payload from the coumarin compound**s**. The gradual decrease in fluorescence intensity within 1 h can be attributed to the photochemical transformation of the coumarin chromophore upon the PMB releasing. These distinct spectroscopic features provide additional support for the proposed photo‐elimination mechanism, which involves more complex electronic transitions compared to the straightforward photolysis of compound **8**.

As shown in Figure 4d, by plotting the fluorescence decrement against irradiation time, a first‐order kinetic trend can be observed, similar to the kinetic profiles obtained from the HPLC analysis of PMB release. This congruence suggests that the fluorescence intensity decay and the PMB releasing are directly coupled and governed by the same underlying photochemical process. The rate‐determining step, likely involving the formation of the zwitterionic intermediate or the subsequent deprotonation step, appears to be the same for both the PMB release and the fluorescence intensity decay processes. Furthermore, we observe that compound **5** exhibits a noticeably faster rate of fluorescence reduction upon irradiation compared to **7**. This difference in kinetics can be attributed to the nature of the leaving groups associated in the fused coumarins. The compound **5** and **7** possess an acetate and PMB group as the leaving group, respectively; the smaller Ac group is more favorable for the photodissociation due to reduced steric hindrance and greater instability compared to the PMB group. This facilitated departure of the leaving group can accelerate the overall reaction rate. Consequently, the difference in the photolytic process can be simply resolved by fluorescence analysis, suggesting a faster uncaging rate by manifested as a more rapid decrease in fluorescence intensity.

**Figure 5:**
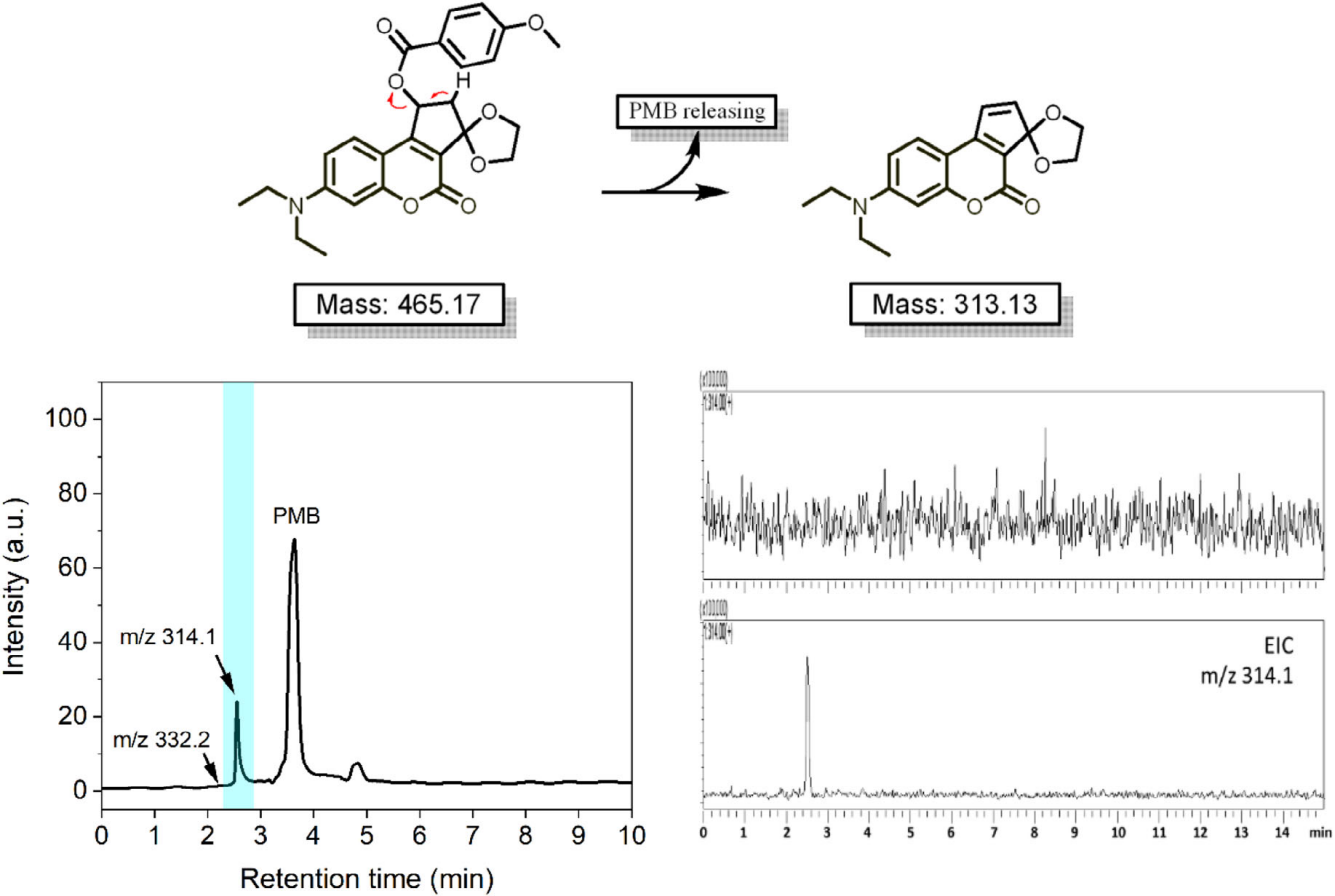
LC‐MS analysis of the photoproduct from compound **7** after 12 h irradiation. The chromatogram was first monitored using a UV–vis detector (*λ* = 250 nm), and subsequent extracted ion chromatogram (EIC) analysis was used to track specific molecular weight of m/z = 314.1, confirming the identity of the photoproduct of a cyclopentene‐fused coumarin structure (retention time = 2.5 min).

The photolytic reaction in methanol/PBS solutions under light excitation was further validated by LC‐MS analyses. As illustrated in Figure 5, upon 12 h irradiation, the HPLC chromatogram monitored by UV–vis detector (*λ* = 250 nm) revealed two major peaks: the PMB payload at retention time 3.5 min and a photoproduct peak at 2.5 min. The PMB was previously confirmed by the standard compound in the reaction kinetics analysis; the identity of the unknown photoproduct was further confirmed through extracted ion chromatogram (EIC) monitoring, which revealed a characteristic signal at m/z 314.1, consistent with the [M + H]^+^ ion of the proposed cyclopentene‐fused structure. Additionally, a tiny signal at 2.1 min was only detectable through EIC analysis at m/z 332.2, which can be attributed to the hydrated species formed during the MS analysis process. This mass spectrometric evidence strongly supports our proposed photo‐elimination mechanism. The formation of this cyclopentene‐fused coumarin photoproduct bearing an external C═C bond, rather than the conventional photo‐substitution product, accounts for the distinct changes observed in the UV–vis absorption and fluorescence spectra after light excitation.

## 3 Conclusions

In this study, we successfully developed a one‐pot Heck–Aldol annulation strategy for synthesizing cyclopentane‐fused coumarin derivatives with photolabile properties. The photophysical investigations revealed that the cyclopentane‐fused structure significantly influences the absorption and emission properties, causing blue‐shifted spectra and reduced extinction coefficients compared to nonfused analogs. Our photolytic studies demonstrated a distinct photo‐elimination mechanism, contrasting with the conventional photo‐S_N_1 pathway of typical coumarin photocages. This mechanism involves heterolytic C─O bond cleavage followed by β‐proton elimination, as confirmed by LC‐MS analysis. The solvent‐dependent kinetics, with accelerated release rates in PBS buffer, provide further support for our proposed mechanistic pathway. We also established that fluorescence spectroscopy offers a convenient method for monitoring the photochemical reaction progress in real time. The direct correlation between fluorescence intensity decrease and photoinduced payload release provides visual feedback of the uncaging process. Despite lower photolysis quantum yields than reference compounds, these cyclopentane‐fused coumarins show promise for applications requiring controlled photorelease of bioactive molecules due to their unique photochemical behavior and moderate fluorescence quantum yields.

## 4 Experimental Section

### 4.1 General Information

All reactions were conducted in flame‐dried glassware, under nitrogen atmosphere, unless mentioned. Tetrahydrofuran, acetonitrile, toluene and dichloromethane were purified and dried from a safe purification system containing activated Al_2_O_3_. All reagents obtained from commercial sources were used without purification. Flash column chromatography was carried out on silica gel 60 (23–60 mesh, E. Merck). TLC was performed on pre‐coated glass plates of Silica Gel 60 F254 (0.25 mm, E. Merck). ^1^H, and ^13^C‐NMR were measured by using Varian Mercury‐400 MHz, Bruker DMX‐400 MHz, and JEOL ECZ400S/L1 400 MHz spectrometers. Chemical shift was reported as *δ* values in ppm, and calibrated by using residual undeuterated solvent (CDCl_3_ (7.26 ppm)) as internal reference for ^1^H NMR and the deuterated solvent (CDCl_3_ (77.0 ppm)) as internal standard for ^13^C NMR. Coupling constants were reported in Hz, and multiplicities were indicated as follows: s (singlet); d (doublet); t (triplet); m (multiplet). Mass spectra for materials characterization were analyzed on a Finnigan LTQ‐OrbitrapxL instrument with an electrospray ionization (ESI) source. A solution of compounds (at 100 µM) in MeOH/H_2_O (9:1 v/v) at 25 °C in a 2 mL quartz cuvettes was exposed to a LUMOS 43 LED equipment using the 405 nm irradiation mode (Atlas Photonics Inc., Fribourg, Switzerland). The reaction was monitored by UV and aliquots of samples (20 µL) were further analyzed by HPLC to determine the percentage of released payload using a calibration curve. UV–vis absorption spectra were recorded on a Thermo Genesys 10S UV–vis spectrometer (ThermoFisher Scientific, Waltham, MA, USA). Fluorescence spectra were recorded on a Hitachi F‐2700 Spectrometer (Hitachi High‐Tech., Tokyo, Japan). HPLC was performed on a JASCO instrument equipped with a UV–970 UV–vis detector covering the wavelengths from 200–900 nm. The HPLC analysis was conducted at 25 °C using Dr. Maisch ReproSil‐100 C18 column (250 × 4.6 mm, reverse phase with 5 um porous spherical silica). A solvent mixture of methanol (90%) and deionized water (10%) was used as a mobile phase and the flow rate was maintained at 1.0 cm^3^/min. The LC‐MS analysis was performed on a Shimadzu LCMS‐8040 triple quadrupole mass spectrometer (Kyoto, Japan) equipped with an electrospray ionization (ESI) source operating in both positive and negative ion modes. Chromatographic separation was achieved using an Agilent 5 HC‐C18(2) column (150 × 4.6 mm, 5 µm particle size) maintained at 40 °C. The injection volume was 2 µL, with a mobile phase consisting of deionized water (10%) and methanol (90%). Extracted ion chromatogram (EIC) analyses with selected ion monitoring were recorded using Shimadzu LabSolutions software.

### 4.2 Synthetic Procedures and Characterization

#### 4.2.1 3‐Bromo‐7‐(Diethylamino)‐2‐Oxochromene‐4‐Carbaldehyde (**2**)

To a solution of **1** (800 mg, 3.26 mmol, 1.0 equiv.) in MeCN (30 mL) was added NBS (650 mg, 3.59 mmol, 1.1 equiv.) and NH_4_OAc (25 mg, 0.033 mmol) under an Ar atmosphere. The reaction mixture immediately turned dark brown and was stirred for 30 min, then poured into water and extracted with EtOAc. The combined organic layers were dried over MgSO4, filtered and concentrated under reduced pressure. Purification by flash column chromatography on silica gel (EtOAc/hexanes = 1:2) afforded **2** (1.08 g, 99%) as a brown solid. *R*_f_ = 0.3 (EtOAc/hexanes = 1:2); ^1^H NMR (400 MHz, CDCl_3_) δ 10.33 (s, 1H), 7.91 (d, *J* = 9.3 Hz, 1H), 6.59 (dd, *J* = 9.3, 2.7 Hz, 1H), 6.46 (d, *J* = 2.6 Hz, 1H), 3.40 (q, *J* = 7.1 Hz, 4H), 1.20 (t, *J* = 7.1 Hz, 6H); ^13^C{^1^H} NMR (101 MHz, CDCl_3_) δ 192.8, 158.1, 155.6, 150.9, 141.8, 126.5, 109.8, 109.4, 104.5, 97.2, 44.8, 12.3.

#### 4.2.2 7′‐(Diethylamino)‐1′‐Hydroxyspiro[1,3‐Dioxolane‐2,3′‐Cyclopenta[c]Chromene]‐4′‐one (**3a**)

A two‐neck round‐bottom flask equipped with a stir bar was charged with **2** (450 mg, 1.39 mmol), Pd(OAc)_2_ (3.5 mg, 1 mol%), and dppp (9 mg, 0.021 mmol). The flask was degassed three times with N_2_, then ethylene glycol (15 mL), *n*‐butyl vinyl ether (540 µL, 4.2 mmol), and Et_3_N (300 µL, 2.1 mmol) were added sequentially via syringe. The reaction mixture was stirred vigorously at 115 °C for 16 h. After cooling to room temperature, the mixture was diluted with EtOAc and water. The aqueous layer was extracted with EtOAc (3 × 10 mL), and the combined organic layers were washed with brine, dried over MgSO4, filtered, and concentrated under reduced pressure. Purification by flash column chromatography on silica gel (EtOAc/hexanes = 1:1) afforded **3a** (64 mg, 14%) as a brown oil. *R*_f_ = 0.18 (EtOAc/hexanes = 1:1); ^1^H NMR (400 MHz, CDCl_3_) δ 8.00 (d, *J* = 9.2 Hz, 1H), 6.60 (dd, *J* = 9.3, 2.6 Hz, 1H), 6.56 (s, 1H), 6.50 (d, *J* = 2.5 Hz, 1H), 4.51–4.47 (m, 1H), 4.00–3.96 (m, 1H), 3.42 (dd, *J* = 14.2, 7.1 Hz, 6H), 1.21 (t, *J* = 7.1 Hz, 9H); ^13^C{^1^H} NMR (101 MHz, CDCl_3_) δ 164.8, 157.0, 150.8, 142.5, 127.6, 124.8, 111.2, 109.2, 104.9, 99.9, 97.6, 67.4, 60.8, 44.8, 30.2, 29.7, 29.6, 12.4; HRMS (ESI) *m/z* [M + H]^+^ calcd for C_18_H_20_NO_5_ 330.1341, found 330.1342.

#### 4.2.3 [7′‐(Diethylamino)‐4′‐Oxospiro[1,3‐Dioxolane‐2,3′‐Cyclopenta[c]Chromene]‐1′‐yl] Acetate (**4**)

To a solution of **3a** (28 mg, 0.085 mmol) in CH_2_Cl_2_ (2 mL) were added Et_3_N (40 µL, 0.25 mmol) and Ac_2_O (18 mg, 0.17 mmol). The reaction mixture was stirred for 3 h under N_2_ atmosphere at room temperature, then diluted with water and extracted with EtOAc. The organic layer was dried over MgSO4, filtered, and concentrated under reduced pressure to afford **4** (31 mg, 99%) as a brown oil without further purification. *R*_f_ = 0.55 (EtOAc/hexanes = 1:1); ^1^H NMR (400 MHz, CDCl_3_) δ 7.99 (d, *J* = 9.2 Hz, 1H), 6.60 (dd, *J* = 9.2, 2.6 Hz, 1H), 6.54 (s, 1H), 6.50 (d, *J* = 2.6 Hz, 1H), 4.57–4.54 (m, 2H), 4.42–4.39 (m, 2H), 3.42 (dd, *J* = 14.2, 7.1 Hz, 7H), 2.11 (s, 3H), 1.21 (t, *J* = 7.1 Hz, 12H); ^13^C{^1^H} NMR (101 MHz, CDCl_3_) δ 170.8, 164.4, 161.6, 157.1, 150.8, 142.2, 127.6, 111.5, 109.2, 104.9, 99.9, 97.6, 63.6, 61.8, 44.8, 29.7, 20.8, 12.4; HRMS (ESI) *m/z* [M + H]^+^ calcd for C_20_H_22_NO_6_ 372.1447, found 372.1438.

#### 4.2.4 [7′‐(Diethylamino)‐4′‐Oxospiro[1,3‐Dioxolane‐2,3′‐1,2‐Dihydrocyclopenta[c]Chromene]‐1′‐yl] Acetate (**5**)

To a solution of **4** (24 mg, 0.065 mmol) in MeOH (20 mL) was added Pd(OH)_2_/C (2.4 mg, 10 wt%). The reaction mixture was stirred for 2 h under H_2_ atmosphere of 5 bar, then diluted with water and extracted with EtOAc. The organic layer was dried over MgSO4, filtered, and concentrated under reduced pressure. Purification by flash column chromatography on silica gel (EtOAc/hexanes = 1:2) afforded **5** (7 mg, 29%) as a yellow solid. *R*_f_ = 0.32 (EtOAc/hexanes = 1:2); ^1^H NMR (400 MHz, CDCl_3_) δ 7.10 (d, *J* = 8.6 Hz, 1H), 6.40 (dd, *J* = 8.6, 2.6 Hz, 1H), 6.33 (d, *J* = 2.6 Hz, 1H), 4.29 (dd, *J* = 5.4, 4.1 Hz, 2H), 4.25–4.21 (m, 2H), 3.85 (dd, *J* = 6.3, 2.9 Hz, 1H), 3.33 (q, *J* = 7.1 Hz, 4H), 3.08 (dd, *J* = 16.2, 2.9 Hz, 1H), 2.80 (dd, *J* = 16.2, 6.4 Hz, 1H), 2.03 (s, 3H), 1.15 (t, *J* = 7.1 Hz, 6H); ^13^C{^1^H} NMR (101 MHz, CDCl_3_) δ 171.6, 170.9, 166.9, 152.9, 149.1, 129.1, 107.8, 104.7, 99.7, 63.1, 61.8, 44.6, 40.5, 31.9, 29.8, 20.8, 12.5; HRMS (ESI) *m/z* [M + H]^+^ calcd for C_20_H_24_NO_6_ 374.1604, found 374.1609.

#### 4.2.5 [7′‐(Diethylamino)‐4′‐Oxospiro[1,3‐Dioxolane‐2,3′‐Cyclopenta[c]Chromene]‐1′‐yl] 4‐Methoxybenzoate (**6**)

To a solution of **3a** (20 mg, 0.061 mmol) in CH_2_Cl_2_ (5 mL) were added DMAP (2 mg, 10 wt%) and PMB anhydride (19.4 mg, 0.067 mmol). The reaction mixture was stirred for 24 h under N_2_ atmosphere at room temperature, then diluted with water and extracted with EtOAc. The organic layer was dried over MgSO4, filtered, and concentrated under reduced pressure. Purification by flash column chromatography on silica gel (EtOAc/hexanes = 1:3) to afford **6** (15 mg, 53%) as a brown oil. *R*_f_ = 0.57 (EtOAc/hexanes = 1:1); ^1^H NMR (400 MHz, CDCl_3_) δ 8.02 (d, *J* = 9.0 Hz, 2H), 7.95 (d, *J* = 9.5 Hz, 1H), 6.93 (d, *J* = 9.2 Hz, 2H), 6.54 (s, 1H), 6.51–6.44 (m, 2H), 4.73–4.60 (m, 4H), 3.86 (s, 4H), 3.39 (q, *J* = 7.1 Hz, 4H), 1.19 (t, *J* = 7.1 Hz, 6H). ^13^C{^1^H} NMR (101 MHz, CDCl_3_) δ 165.9, 164.5, 163.6, 161.5, 157.0, 150.8, 142.4, 131.8, 127.6, 121.9, 113.7, 111.4, 109.1, 104.9, 97.6, 63.8, 62.0, 55.4, 44.7, 29.7, 12.4; HRMS (ESI) *m/z* [M‐H]^‐^ calcd for C_26_H_24_NO_7_ 462.1553, found 462.1536.

#### 4.2.6 [7′‐(Diethylamino)‐4′‐Oxospiro[1,3‐Dioxolane‐2,3′‐1,2‐Dihydrocyclopenta[c]Chromene]‐1′‐yl] 4‐Methoxybenzoate (**7**)

To a solution of **6** (15 mg, 0.032 mmol) in MeOH (20 mL) was added Pd(OH)_2_/C (1.5 mg, 10 wt%). The reaction mixture was stirred for 2 h under H_2_ atmosphere of 5 bar, then diluted with water and extracted with EtOAc. The organic layer was dried over MgSO4, filtered, and concentrated under reduced pressure to afforded **7** (12.8 mg, 87%) as a yellow oil without further purification. *R*_f_ = 0.54 (EtOAc/hexanes = 1:1); ^1^H NMR (400 MHz, CDCl_3_) δ 7.92 (d, *J* = 9.0 Hz, 2H), 7.07 (dd, *J* = 8.8, 0.5 Hz, 1H), 6.90 (d, *J* = 9.0 Hz, 2H), 6.30–6.25 (m, 2H), 4.52–4.35 (m, 5H), 3.86 (s, 3H), 3.27 (q, *J* = 7.1 Hz, 4H), 3.08 (dd, *J* = 16.2, 3.2 Hz, 1H), 2.80 (dd, *J* = 16.2, 6.3 Hz, 1H), 1.11 (t, *J* = 7.1 Hz, 6H); ^13^C{^1^H} NMR (101 MHz, CDCl_3_) δ 171.5, 166.8, 165.9, 163.5, 152.7, 148.9, 131.8, 128.9, 122.0, 113.6, 107.8, 107.8, 99.7, 63.1, 62.1, 55.4, 44.4, 40.4, 31.8, 29.7, 12.4; HRMS (ESI) *m/z* [M‐H]^‐^ calcd for C_26_H_26_NO_7_ 464.1709, found 464.1704.

## Supporting Information

The reaction optimization for compound 3a and ^1^H/^13^C NMR spectra for materials characterization are available free of charge in the Electronic Supporting Information (ESI).

## Conflict of Interests

The authors declare no conflicts of interest.

## Supporting information



Supporting Information

## Data Availability

The data that support the findings of this study are available from the corresponding author upon reasonable request.
