# Supplementary material for: Engineering Photolabile Cyclopentane‐Fused Coumarins: From One‐Pot Synthesis to Mechanistic Study for Visible Light‐Triggered Photolysis
Source: Chem Asian J. 2025 Jul 29;20(19):e00760. doi: 10.1002/asia.202500760 (PMC12498183; doi:10.1002/asia.202500760)
Supplement: Supplementary file 1 — Supporting Information [file ASIA-20-e00760-s001.docx]

**Supporting Information**

**Engineering Photolabile Cyclopentane-Fused Coumarins: From One-Pot Synthesis to Mechanistic Study for Visible Light-Triggered Photolysis**

Jau-Tien Lin,^a^ Hsuan-Yu Lin,^a^ Tai-Chung Lo,^b^ Chih-Ling, Lin,^a^ Yi-Hsien Li,^a^ Wei-Hao Wu,^a^ Ting-Yi, Hsieh,^a^ Shun-Yuan Luo,*^b^ and Chih-Chien Chu,*^ac^

^a^Department of Medical Applied Chemistry, Chung Shan Medical University, Taichung 402, Taiwan

^b^Department of Chemistry, National Chung Hsing University, Taichung 403, Taiwan

^c^Department of Medical Education, Chung Shan Medical University Hospital, Taichung 402, Taiwan

*Corresponding Author: Prof. Dr. Chih-Chien Chu

TEL: +886-4-36097617; e-mail: **jrchu@csmu.edu.tw**

-
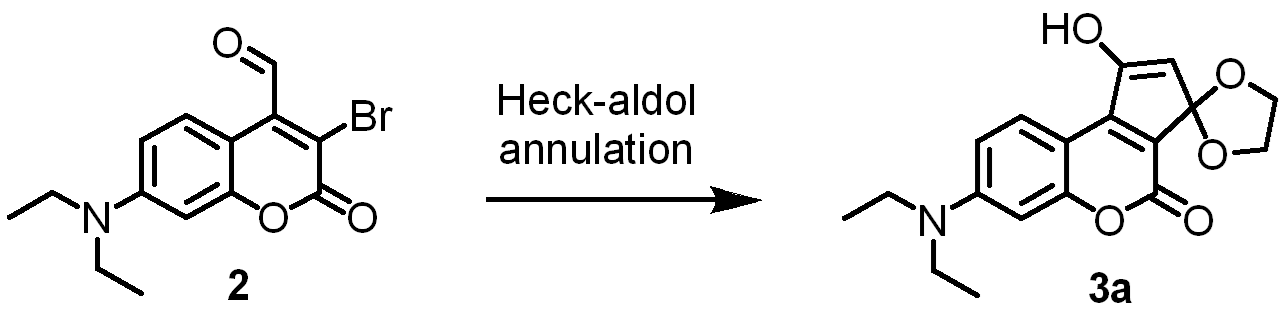
Optimization for the synthesis of compound **3a**

**Pd(OAc)_2_-dppp**

| **Heck-aldol annulation for compound 3a** | | | | |
| --- | --- | --- | --- | --- |
| entry | Temp (^o^C) | Reagent^[a]^ | Solvent^[b]^ | yield (%)^[c]^ |
| **1** | 80 | VBE | EG | 7 |
| **2** | 60 | VBE | EG | 6 |
| **3** | 100 | VBE | EG | 13 |
| **4** | 115 | VBE | EG | 14 |
| **5** | 115 | VBE/AcOH | DMF | 0 |
| **6** | 115 | 2-ethoxyethanol | EG | 13 |
| [a] VBE: vinyl butyl ether. [b] EG: ethylene glycol. [c] Isolated yields | | | | |

**
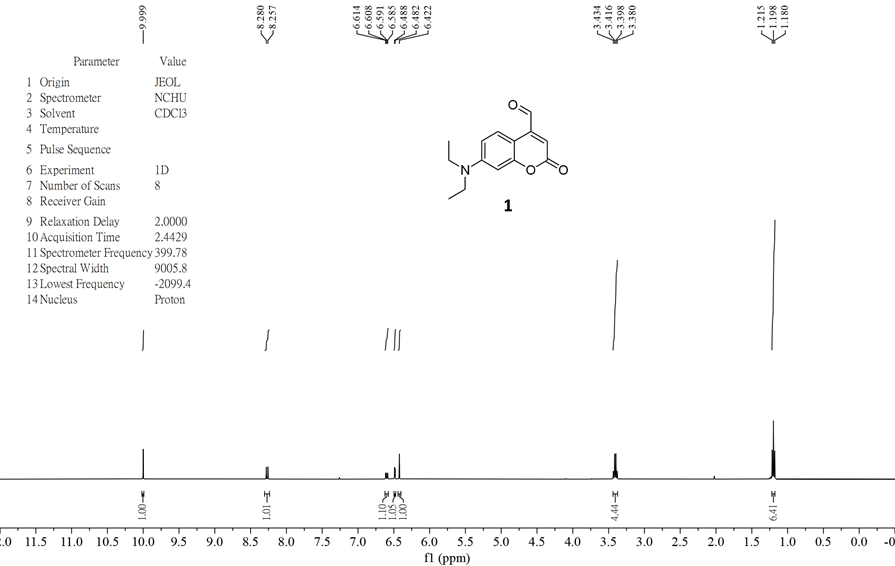
**

**
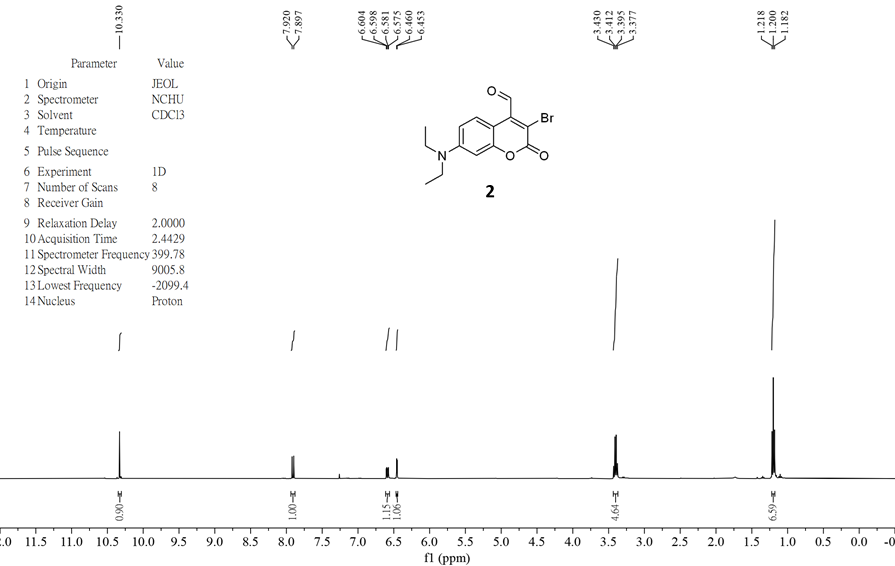
**

**
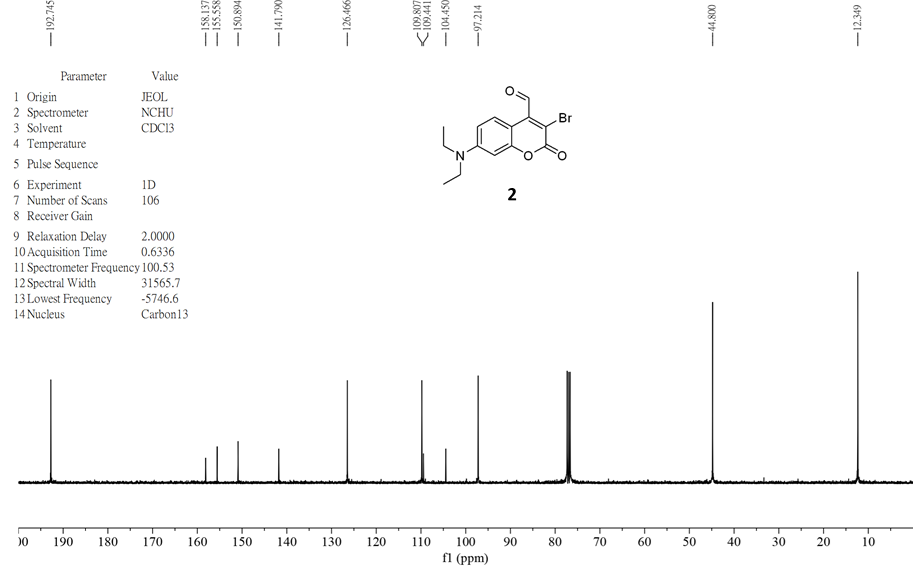
**

**
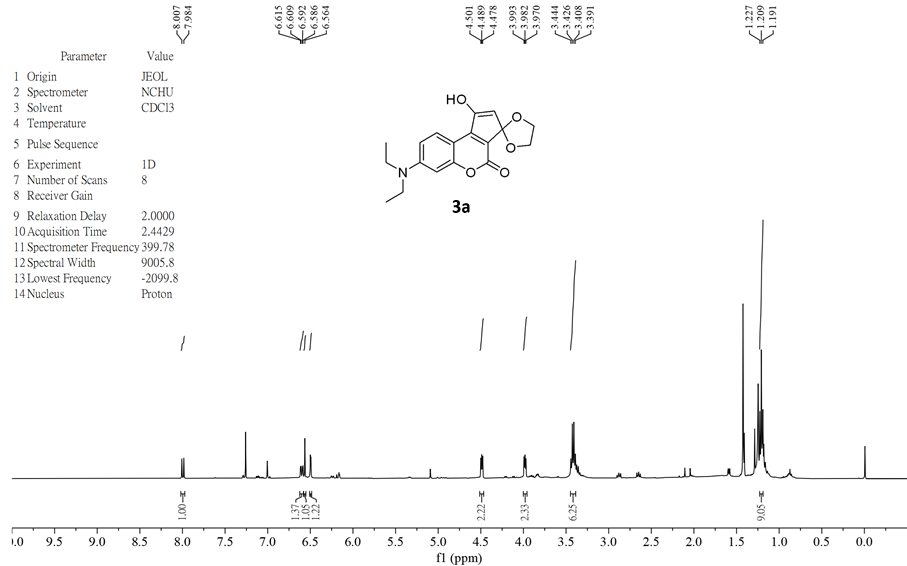
**

**
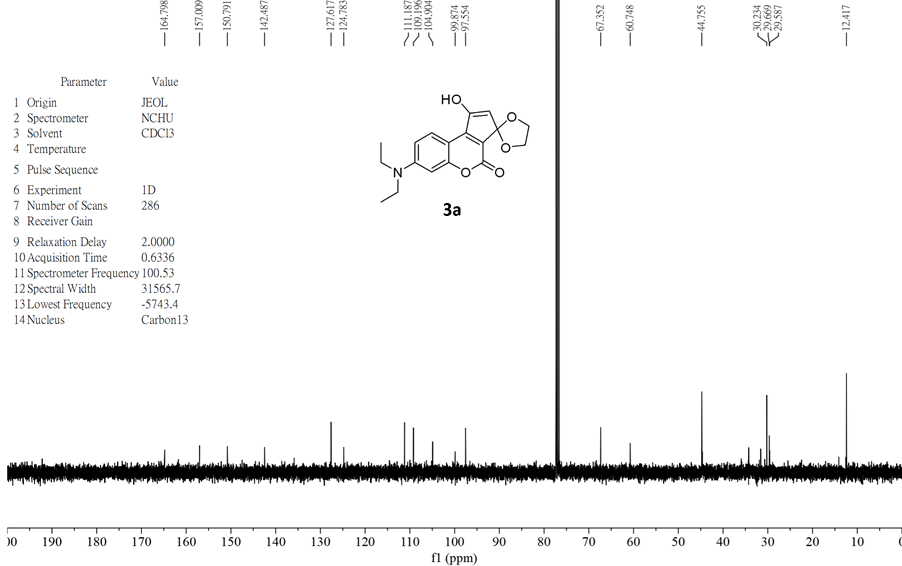
**

**
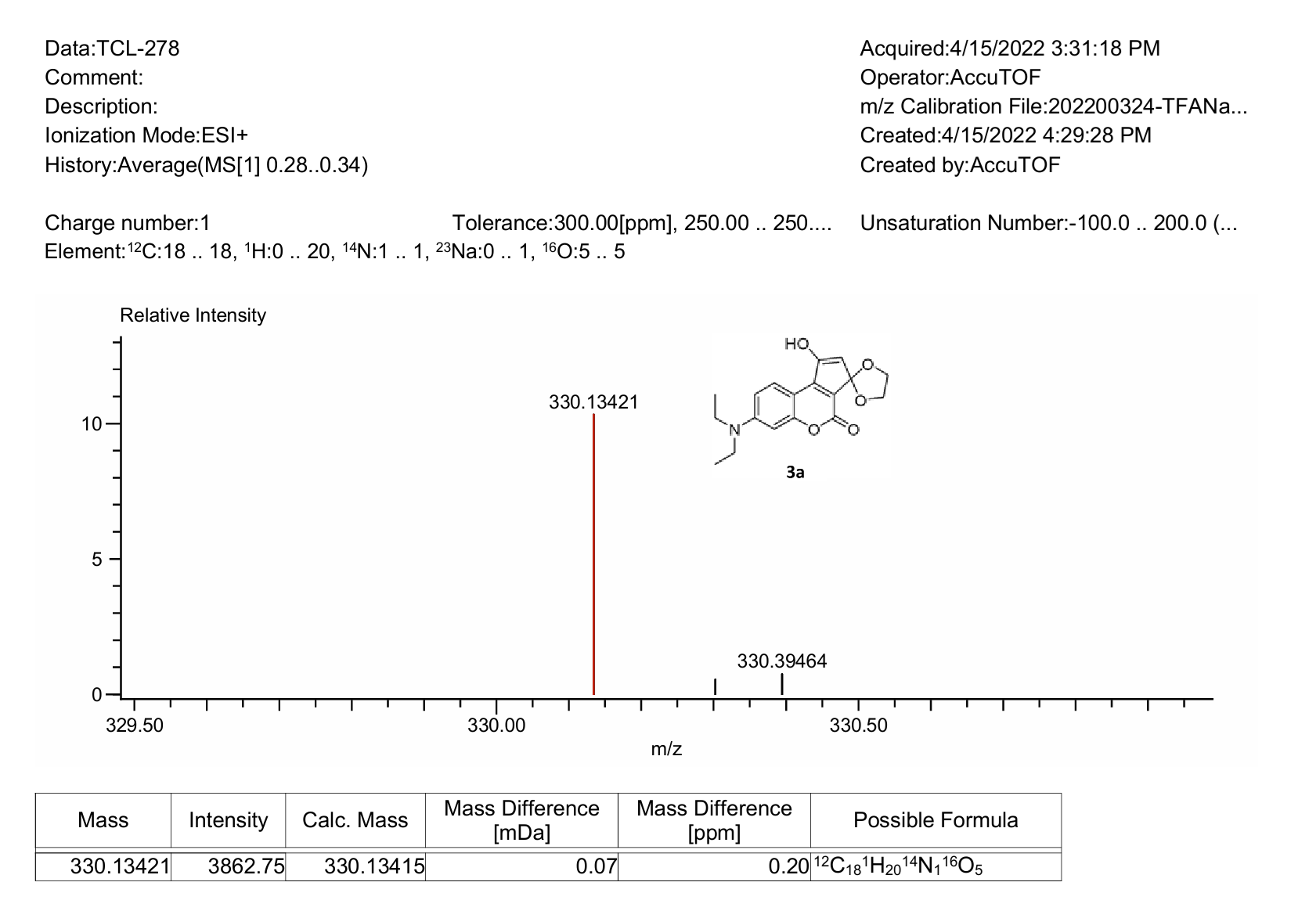
**

**
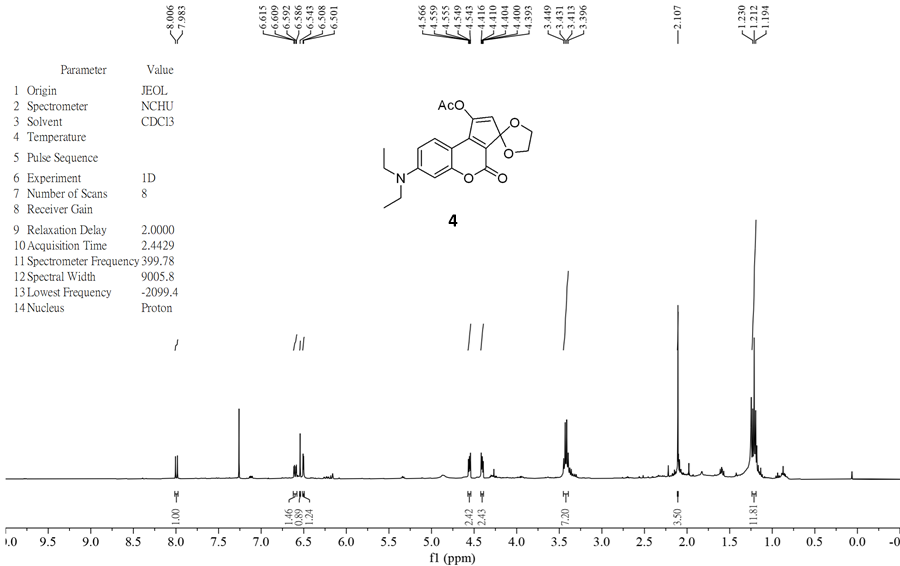
**

**
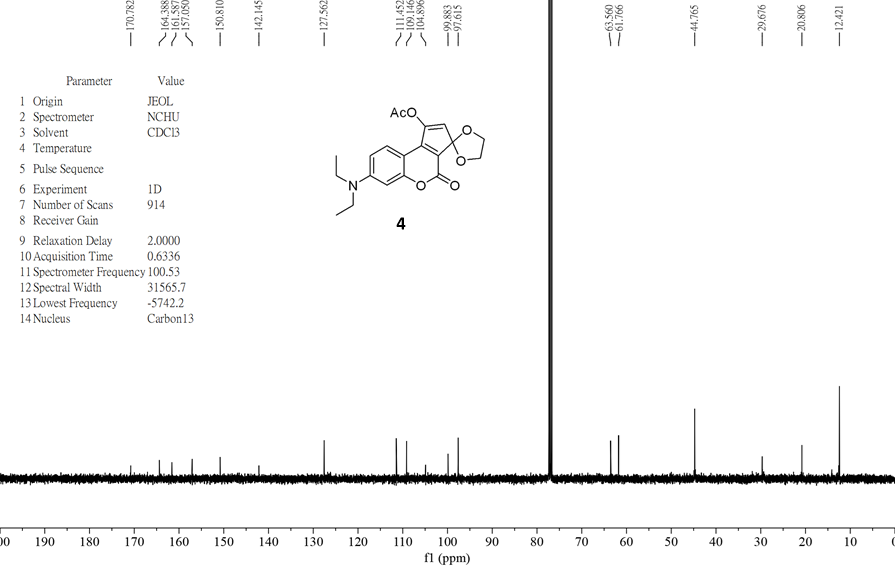
**

**
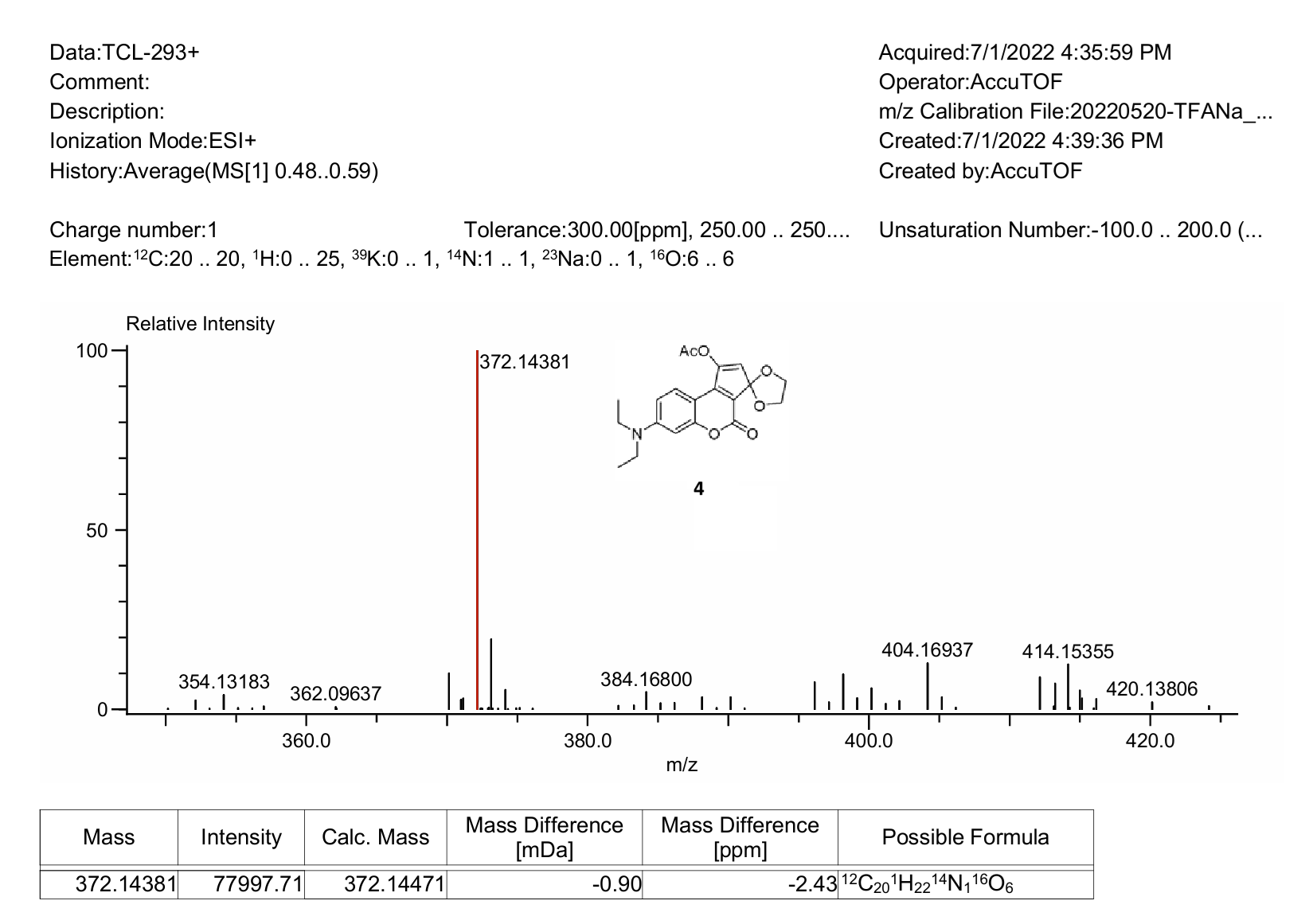
**

**
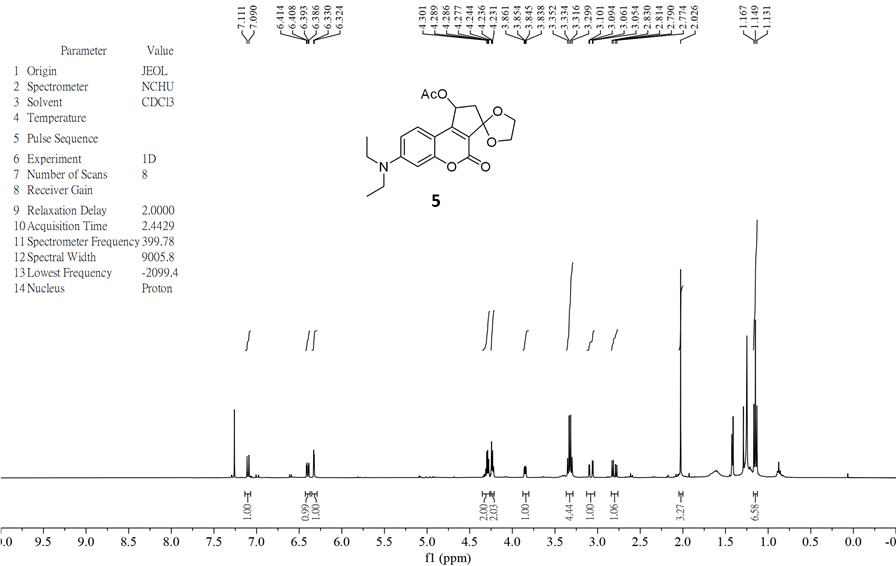
**

**
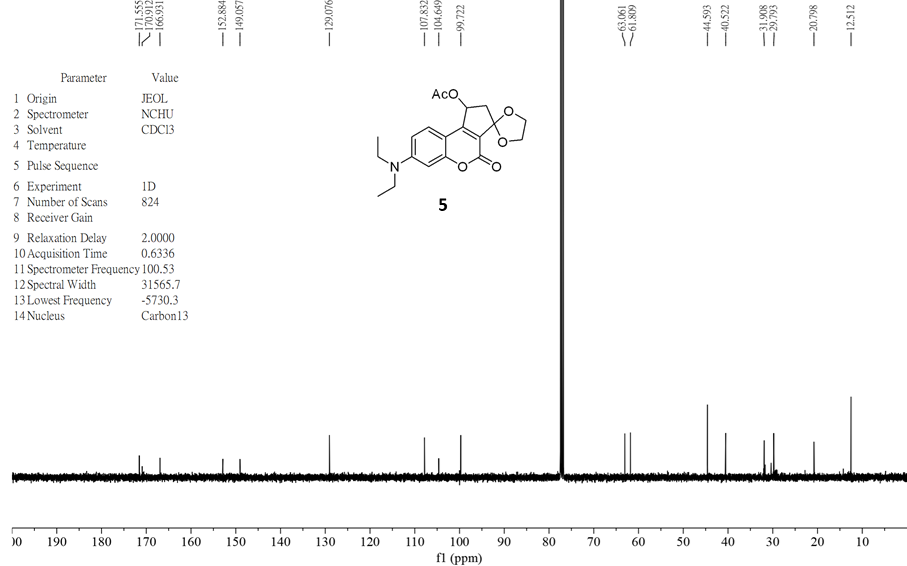
**

**
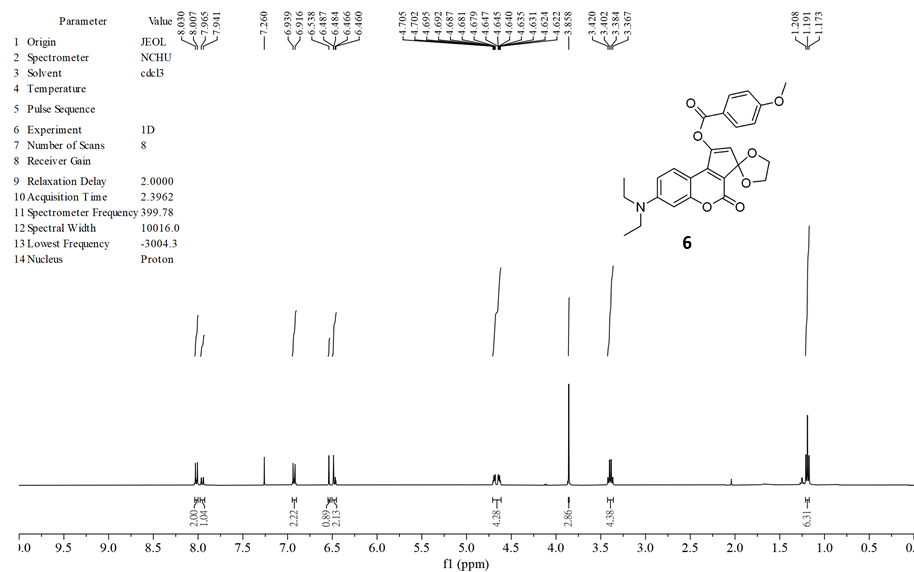
**

**
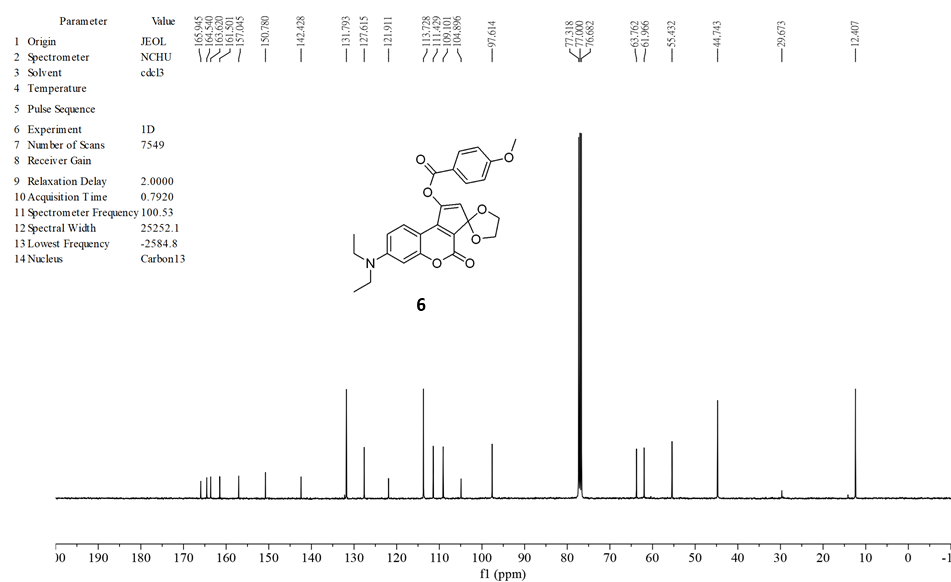
**

**
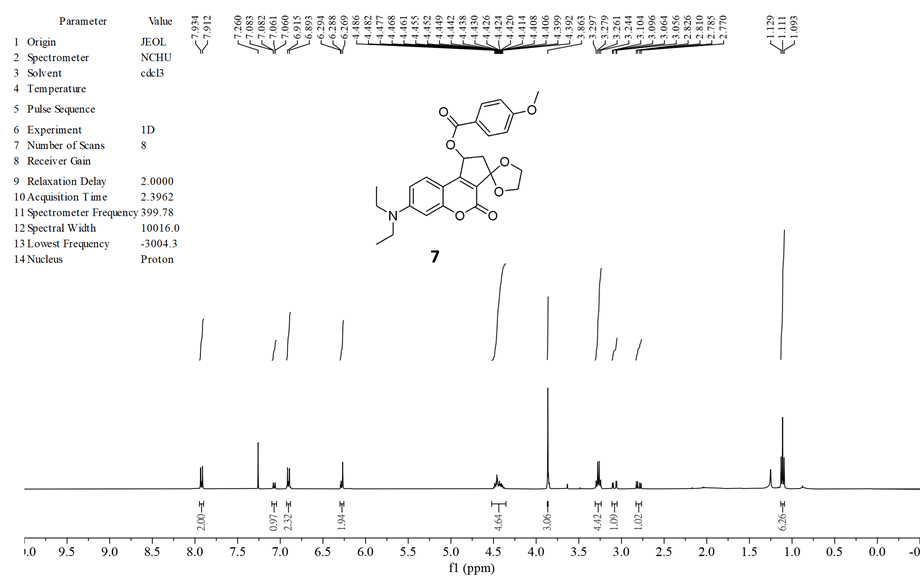
**

**
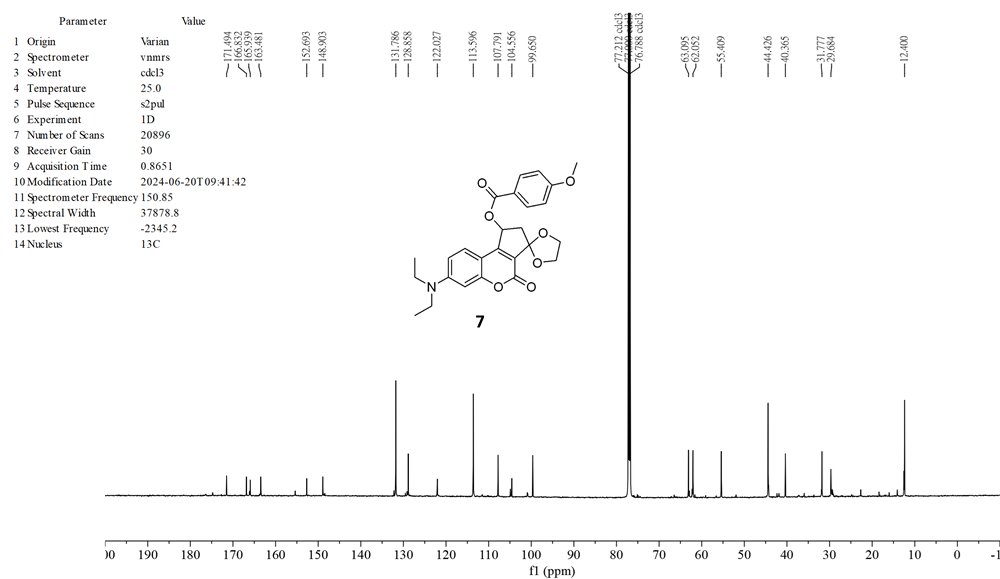
**

**
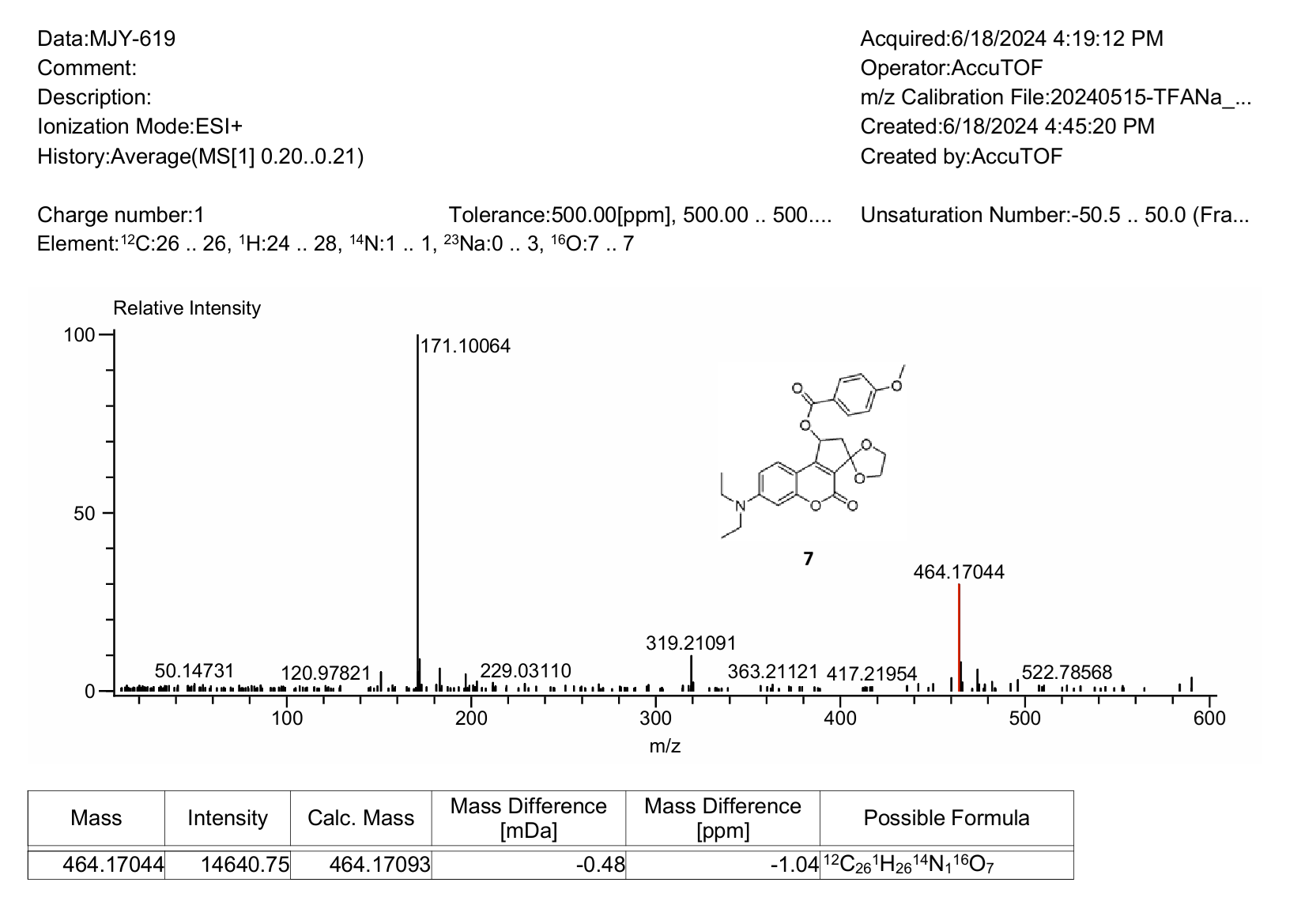
**
